# Associations between cerebellar development and autistic traits during adolescence: a population-based cohort study

**DOI:** 10.1192/j.eurpsy.2024.312

**Published:** 2024-08-27

**Authors:** C. Morimoto, S. Koike

**Affiliations:** ^1^Fuculty of Allied Health Schience, Niigata University of Rehabilitation, Niigata; ^2^Department of Neuropsychiatry, Graduate School of Medicine, University of Tokyo, Tokyo, Japan

## Abstract

**Introduction:**

Brain maturation is associated with adolescent socio-cognitive development. The lateral posterior region of the cerebellum plays a critical role in higher cognitive processes, and deviations of this region are associated with autism-related behaviors. Hence, it is plausible that developmental changes in this region of the cerebellum during adolescence are different along a variation in autistic traits. Additionally, its difference may be moderated by parental age at birth and weight growth during infancy, which have effects on brain development.

**Objectives:**

The aim of this study was two folds: (1) to test whether cerebellar development during adolescence is different along a variation in autistic traits (2) to test whether parental age at birth and weight growth during infancy moderate the results of (1).

**Methods:**

Longitudinal study was conducted over a 6-year period with 256, 230 and 187 participants ranging from 10.5 to 17.6 years, observing adolescent development respectively at 2-year time periods. We undertook a detailed investigation into differences in the lateral posterior region of the cerebellum volume. The 50-item Autism-Spectrum Quotient (AQ) was rated by primary parents. Weight growth and parental age were evaluated using maternal and child health handbook records. A multiple regression analysis was performed to examine whether AQ subscales, sex, and their interactions affected cerebellar development. Moderation analysis assessed whether parental age and weight growth moderated associations between cerebellar development and autistic traits. All participants provided written informed consent, and the study was approved by the Ethics Committee (No.10069).

**Results:**

Interactions between sex and attention switching and sex and attention to detail were significantly associated with cerebellar development in the bilateral gray matter (GM) and white matter (WM) of Crus I and Crus II (Fig1, 2). Simple slope analyses showed that the slopes of cerebellar development were significant for girls (*p*_FDR_ < 0.001). Although no significant interaction was found between them, the main effect of attention to detail was significantly associated with cerebellar development in WM of VIIB (*p*_FDR_ = 0.006). Further, moderation analysis found that the association between the cerebellar development and autistic traits were significantly moderated by maternal age; the magnitude of its effect was significant for high maternal age in boys (*p*_FDR_ = 0.036, Fig3). Paternal age, early (0-9 months) and late weight growth (4-18 months) also moderated associations between them, however, no significance remained after FDR controlling.

**Image:**

**Figure:**
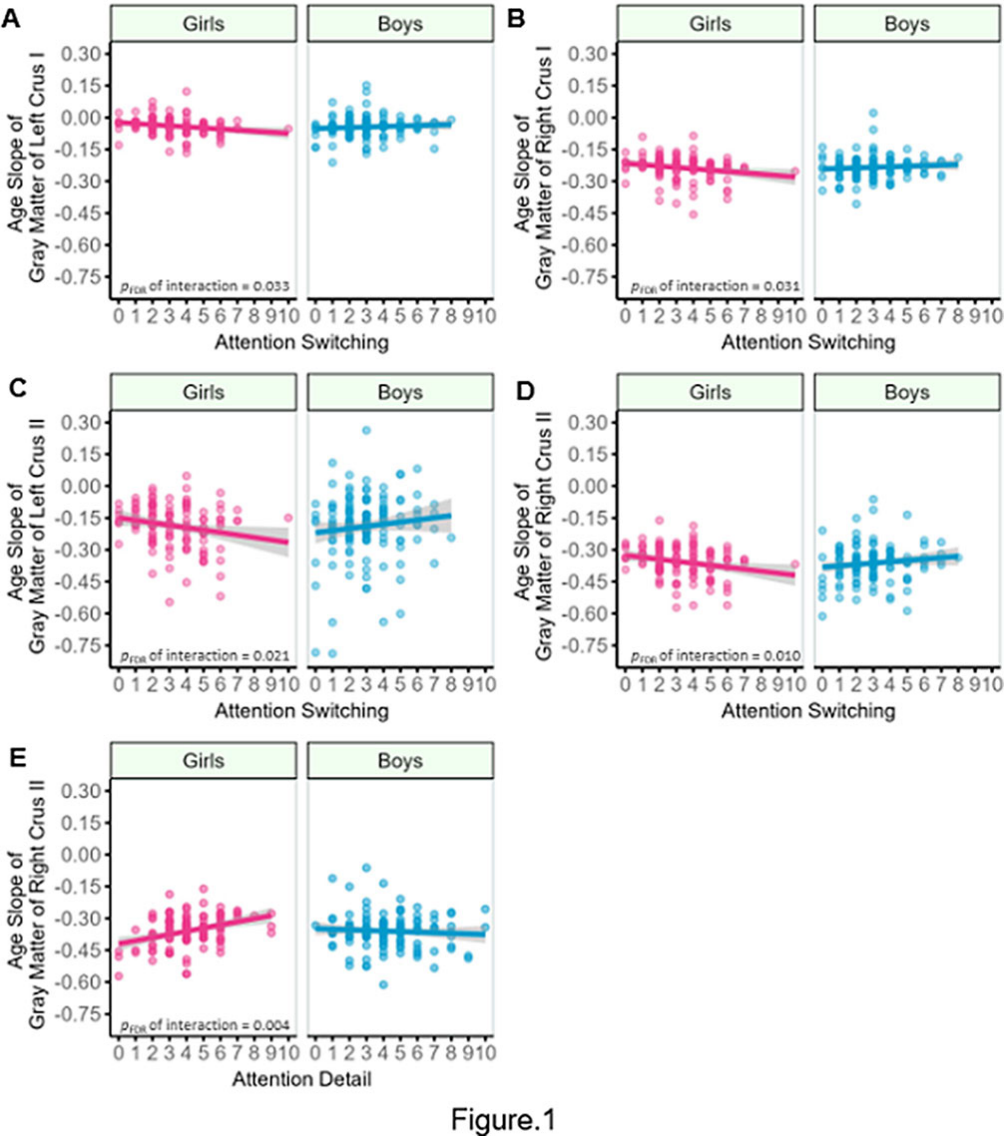

**Image 2:**

**Figure:**
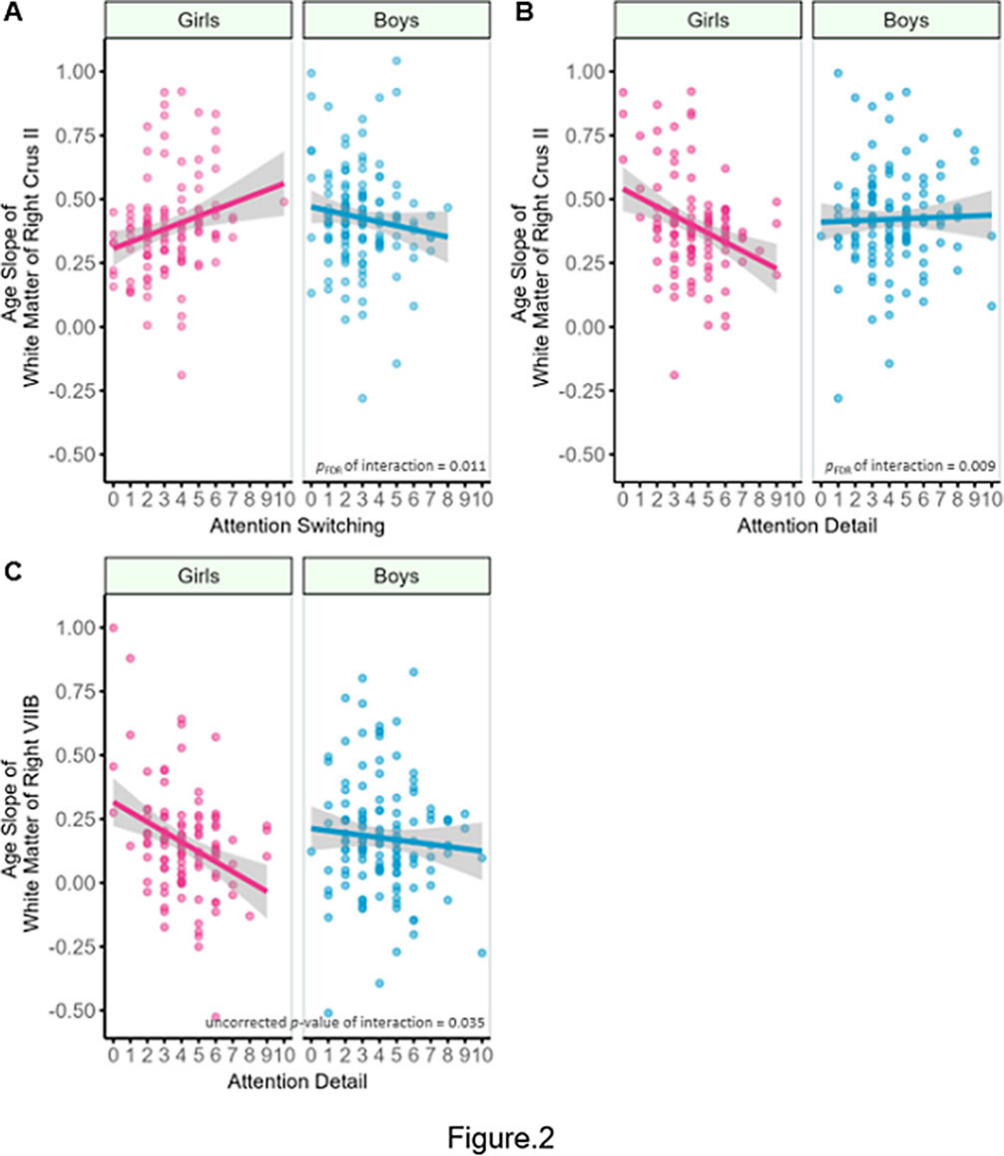

**Image 3:**

**Figure:**
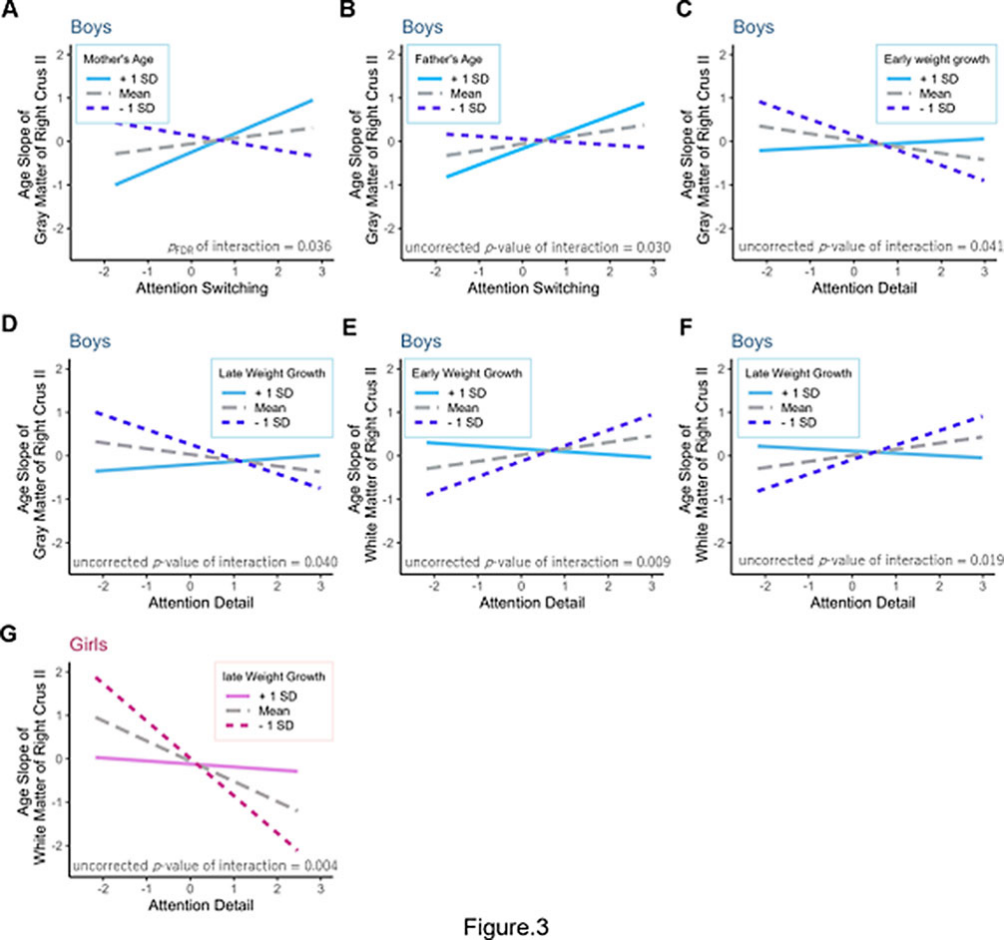

**Conclusions:**

There are significant associations between cerebellar development during adolescence and autistic traits, and its pattern of association can be moderated by parental ages at birth and weight growth during infancy in a cerebellar region- and sex-specific manner.

**Disclosure of Interest:**

None Declared

